# Temporal Decay Loss for Adaptive Log Anomaly Detection in Cloud Environments

**DOI:** 10.3390/s25092649

**Published:** 2025-04-22

**Authors:** Lelisa Adeba Jilcha, Deuk-Hun Kim, Jin Kwak

**Affiliations:** 1 ISAA Laboratory, Department of AI Convergence Network, Ajou University, Suwon 16499, Republic of Korea; jilchalelisa@ajou.ac.kr; 2 ISAA Laboratory, Institute for Computing and Informatics Research, Ajou University, Suwon 16499, Republic of Korea; kimdh1206@ajou.ac.kr; 3 Department of Cyber Security, Ajou University, Suwon 16499, Republic of Korea

**Keywords:** anomaly detection, adaptive detection, cloud computing, log preprocessing, LDF, pretrained language model, temporal decay loss, temporal dependency, zero-shot detection

## Abstract

Log anomaly detection in cloud computing environments is essential for maintaining system reliability and security. While sequence modeling architectures such as LSTMs and Transformers have been widely employed to capture temporal dependencies in log messages, their effectiveness deteriorates in zero-shot transfer scenarios due to distributional shifts in log structures, terminology, and event frequencies, as well as minimal token overlap across datasets. To address these challenges, we propose an effective detection approach integrating a domain-specific pre-trained language model (PLM) fine-tuned on cybersecurity-adjacent data with a novel loss function, Loss with Decaying Factor (LDF). LDF introduces an exponential time decay mechanism into the training objective, ensuring a dynamic balance between historical context and real-time relevance. Unlike traditional sequence models that often overemphasize outdated information and impose high computational overhead, LDF constrains the training process by dynamically weighing log messages based on their temporal proximity, thereby aligning with the rapidly evolving nature of cloud computing environments. Additionally, the domain-specific PLM mitigates semantic discrepancies by improving the representation of log data across heterogeneous datasets. Extensive empirical evaluations on two supercomputing log datasets demonstrate that this approach substantially enhances cross-dataset anomaly detection performance. The main contributions of this study include: (1) the introduction of a Loss with Decaying Factor (LDF) to dynamically balance historical context with real-time relevance; and (2) the integration of a domain-specific PLM for enhancing generalization in zero-shot log anomaly detection across heterogeneous cloud environments.

## 1. Introduction

Cloud computing, while offering scalable and on-demand resources, faces significant security threats, including data breaches, unauthorized access, insider threats, and service disruptions caused by configuration flaws or malicious activity [1,2]. These challenges are intensified by the distributed and dynamic nature of cloud infrastructures, which expand the attack surface and render traditional perimeter-based defenses ineffective [2,3]. To address these threats, cloud environments typically adopt a range of countermeasures, including access control mechanisms, encryption, multi-factor authentication, intrusion detection systems (IDS), and continuous monitoring frameworks [1,2]. Despite these efforts, the complexity and scale of modern cloud systems demand more adaptive and fine-grained approaches. System-level telemetry, particularly log data, offers a rich source of information for identifying operational anomalies and latent security threats [3,4,5,6,7]. As such, analyzing these logs in real time becomes essential for detecting abnormal behavior and safeguarding cloud services against both known and emerging threats. Consequently, the reliability of cloud computing environments heavily depends on log-based diagnostics for identifying software malfunctions, performance bottlenecks, and security vulnerabilities. System logs, continuously generated by job schedulers, resource managers, and various application components, encode critical operational states using timestamps, error codes, and runtime metrics [4,5,6]. Although log-based anomaly detection has been extensively studied, challenges emerge when models trained on one dataset are applied to a different log dataset, a scenario commonly referred to as zero-shot or cross-domain detection [7,8,9]. The primary obstacle to cross-domain generalization lies in data drift and evolving logging conventions, which lead to shifts in log structures, terminology, and event frequencies [8,9]. Additionally, minimal token overlaps across datasets exacerbate the difficulty, as log messages from different environments often follow distinct formats and vocabulary, limiting a model’s ability to transfer knowledge across domains (see Section 4).

To address the generalization bottleneck, a viable strategy is to identify a characteristic that remains consistent across heterogeneous environments despite variations in dataset-specific distributions. Prior research has demonstrated that temporal dependency is one such invariant feature of log sequences worth exploiting [10,11,12]. DeepLog [10] and similar works [11,12,13] have successfully employed sequential modeling architectures such as LSTM (Long Short-Term Memory) [11], GRU (Gated Recurrent Unit) [12], and Transformer [13] to capture long-term dependencies between log events, improving anomaly detection performance. These models assume that past events influence future system states, making them particularly effective for learning event sequences in controlled environments. However, while these approaches achieve high accuracy in in-domain settings, their effectiveness significantly degrades when applied to different datasets due to the inherent distributional and structural variations exhibited in these datasets.

A key limitation arises from the preprocessing methods used during the log grouping phase (the common workflow of log anomaly detection is discussed in Section 4). The session ID-based method has proven to be the most reliable technique, outperforming other methods such as fixed window or sliding window-based grouping approaches [7,10]. This method clusters log messages based on unique session identifiers, ensuring that logs from the same execution context remain together [14,15]. However, in supercomputing environments, this approach often results in excessively long sequences, as a single session can last for an extended period and span thousands of log events, some of which may become irrelevant due to system updates, software patches, or reconfigurations. Retaining the entire session increases computational complexity and heightens the risk of overemphasizing outdated events, where anomalies from an old system state impact the detection process, leading to higher false positive rates. Conversely, splitting long sessions into smaller sub-sequences alleviates computational complexity at the cost of disrupting the natural flow of temporal dependencies, potentially discarding vital context necessary for accurate anomaly classification. On the other hand, studies reveal that many supercomputing logs, despite their large size, often contain relatively straightforward anomaly indicators, making simple detection techniques viable for in-domain detection scenarios [7]. Traditional sequence models such as LSTMs can represent long- and short-term dependencies; however, they may overemphasize decayed contexts and prove unnecessarily complex for relatively simple log messages.

The primary contribution of this study is to advance log anomaly detection across heterogeneous datasets by ensuring efficient training and improved generalization performance. To achieve this, we propose an effective detection approach that integrates a novel loss function, Loss with Decaying Factor (LDF), and a domain-specific PLM-based embedding. LDF introduces an exponential time decay mechanism into the model’s training objective, enabling a dynamic balance between historical context and real-time relevance. By maintaining moderate computational complexity and eliminating the overhead of recurrent backpropagation, LDF efficiently models exponential forgetting, thereby accurately capturing the evolving dynamics of cloud computing environments where massive log data are generated within short time frames. Additionally, we employ a domain-specific PLM fine-tuned on cybersecurity-adjacent datasets to mitigate semantic discrepancies arising from variations in log structures, terminology, and event distributions. Hence, the objective of this study can be summarized as: (1) to develop a lightweight yet effective anomaly detection mechanism using a novel loss function (LDF) that models temporal decay; and (2) to leverage domain-specific language models to enhance generalizability across heterogeneous cloud log datasets, particularly under zero-shot conditions.

Rigorous experimental evaluations demonstrate the superior performance of the proposed approach compared to baseline methods across diverse experimental settings. The result highlights that while sequence modelling architectures such as LSTM and Transformer effectively capture long-term dependencies, a simpler loss-level mechanism (LDF) proves sufficient for log datasets characterized by distributional variability and straightforward anomaly patterns. The remainder of this paper is structured as follows: Section 2 introduces foundational concepts necessary for understanding the proposed approach. Section 3 reviews prior research on log anomaly detection, covering both heuristic-based methods and advanced machine learning techniques. Section 4 examines the statistical properties of the datasets used, informing model design and evaluation strategies. Section 5 details the proposed method, including preprocessing steps, domain-specific PLM integration, and the formulation of the Loss with Decaying Factor (LDF). Section 6 presents experimental setup and empirical results, analyzing hyperparameter effects and cross-dataset generalization. Section 7 discusses broader implications, such as adaptive decay strategies and potential future enhancements. Section 8 concludes the study by emphasizing the effectiveness, scalability, and generalizability of the proposed approach in log anomaly detection.

## 2. Related Works

Recent studies have emphasized the growing role of machine learning in cybersecurity, highlighting how various ML algorithms are applied to detect and respond to a wide range of threats across diverse environments [3,4,16,17]. Research on log anomaly detection has similarly evolved from heuristic-driven approaches to sophisticated machine learning and deep neural methods. A standard workflow typically includes log parsing, log grouping, log representation, and detection [11,12]. This section discusses prior work related to these components and their limitations, providing a broader methodological context.

The typical log anomaly detection process begins with log parsing [18,19,20,21], where semi-structured log messages, comprising timestamps, event descriptions, and error codes, are converted into structured templates. Parsing techniques such as pattern mining, clustering, and heuristics-based approaches are common, with the latter proving efficient in real-world scenarios due to its accuracy in handling complex log structures [14,18,19,20]. Log grouping then organizes parsed messages into sequences based on fixed or sliding windows, chronological order, or session IDs [7]. Sliding or fixed windowing segments log into equal-length sequences but risk truncating meaningful temporal dependencies. Session-based grouping, on the other hand, organizes logs based on execution traces and session identifiers, making it a more effective strategy for preserving event continuity [6,7]. However, this method presents its own set of challenges, particularly in cloud and supercomputing environments, where sessions can span thousands of log messages (see Section 4). Consequently, this issue may lead to an overemphasize on outdated events, ultimately degrading model performance by introducing excessive noise into the learning process.

Once logs are grouped, they must be transformed into numerical representations that the underlying detection models can process [6,10,11]. Researchers have developed various neural network-based techniques for log representation, incorporating both static and contextual embeddings. Approaches such as logkey2vec [22] and Template2Vec [11], inspired by word2vec, have been commonly used. However, these methods fall short in capturing the full contextual meaning embedded within log messages. To overcome this gap, recent studies have turned to more advanced models such as GPT, BERT, and RoBERTa, which provide a deeper semantic understanding [13,23,24]. Despite their effectiveness, distributional discrepancies across datasets hinder cross-domain generalization. Moreover, deep learning-based models have been widely explored for the downstream detection head, with various architectures offering different advantages and limitations. Several methods have been developed, including CNN-based [22,25,26], RNN-based [10,11,26], and attention-based [11,13,23,24,26] approaches. DeepLog [10] utilizes LSTMs to model temporal dependencies for real-time anomaly detection at the log entry level, while LogAnomaly [11] enhances this by integrating an attention mechanism with template-based vectorization. LogRobust [13] further refines detection by incorporating Bi-LSTM with attention mechanisms to capture bidirectional dependencies. Meanwhile, PLELog [27] and LogAT [26] address the challenge of data labeling by introducing semi-supervised and transfer learning techniques, respectively. Despite these advancements, a major limitation of these methods is their reliance on static representation models, which may fall short in capturing subtle semantic details, particularly in complex log structures. NeuralLog [23] and LAnoBERT [24] take a BERT-based approach to strengthen the reliability and adaptability of log anomaly detection. However, fully leveraging the contextual semantics within log messages often requires sophisticated architecture such as Transformers, which are designed to handle the high-dimensional vector outputs of these models. This limitation makes them less practical for log datasets, in which anomalies are often simple and easily recognizable.

The existing detection approaches generally suffer from high computational overhead and limited cross-domain and zero-shot generalization due to their reliance on dataset-specific sequential patterns. To address these challenges, researchers have explored more adaptable methods such as SaRLog [8] and MetaLog [9], which leverage globally consistent features. SaRLog [8] employs a BERT-augmented contrastive learning approach, where a fine-tuned BERT model is integrated with a Siamese network using contrastive loss, enabling the model to learn robust log representations with minimal labeled data and improving its generalization in few-shot learning scenarios. MetaLog [9], on the other hand, leverages meta-learning to construct meta-tasks from multiple log datasets, enabling the model to generalize across diverse systems and achieve robust cross-domain detection performance in both zero-shot and few-shot scenarios. To further enhance log representation, the authors introduce the Globally Consistent Semantic Embedding (GCSE) module, which combines pre-trained word embedding with a weighted aggregation mechanism to align log events from different systems into a unified semantic space. In contrast, our approach circumvents the need for complex architectures and extensive training data, while still achieving robust log anomaly detection. When integrated with domain-specific pre-trained language models fine-tuned on cybersecurity datasets, the proposed method provides a resource-efficient and targeted solution ideally suited to environments characterized by distributional variability and straightforward anomaly manifestations. Table 1 summarizes key studies relevant to the proposed method, their main objectives, and the core algorithms or methods they employ, highlighting how each line of work addresses particular challenges in log anomaly detection.

## 3. Preliminaries

This section provides the foundational background and technical underpinnings necessary to understand our proposed method, highlighting the key mathematical concepts and model architectures, such as pretrained language models, CNNs, RNNs, and attention-based networks, commonly employed in log anomaly detection. These preliminaries will help present the rationale behind our design choices and contextualize our contributions in subsequent sections.

### 3.1. Pretrained Language Model-Based Representation

One of the significant advancements in log anomaly detection is the adoption of pretrained language models such as BERT [28] and their variations. These models enhance log analysis by capturing contextual relationships within log messages. BERT, for instance, generates contextual embeddings using a masked language modeling approach, as shown in Equation (1).

(1) $$P\left(x\right)={argMax}_{\theta }\prod_{i=1}^{n}P(x_{i}|x_{<i},x_{>i};\theta )$$

where $x_{i}$ is the $i^{th}$ token in the sequence $x=\left\{x_{1},\;x_{2},\;.\;.\;.\;,\;x_{n}\right\},$ and θ  denotes the model parameters. By learning to predict masked words from surrounding context, BERT captures context-sensitive semantics that go beyond simple n-gram statistics. This enables BERT to develop a nuanced understanding of context, making it particularly effective for extracting semantic features from logs. By leveraging such models, anomaly detection systems can better differentiate between normal and suspicious log patterns, improving accuracy and adaptability across different datasets.

However, general-purpose PLMs may only partially transfer to the log message domain, as they have typically been pretrained on a broad public corpus. The constrained context caused by sparse and domain-specific words exhibited in log datasets (see Section 4) leads to incomplete semantic capture, especially when they involve cloud-specific terminologies such as function calls, error codes, or node states. Such characteristics instantiate the necessity of employing domain-specific representation. Researchers have thus exploited domain-specific PLMs fine-tuned on the cybersecurity-related corpora, enhancing detection accuracy [8].

### 3.2. Deep Learning-Based Log Anomaly Detection

#### 3.2.1. CNN-Based Detection Approaches

Convolutional Neural Networks (CNNs) can learn local feature patterns from log sequences, especially if logs are arranged as 1D or 2D matrices [22,25,26]. If a window of log keys is mapped to vector embeddings, $\left\{v_{1},\;v_{2},\;.\;.\;.\;,\;v_{n}\right\}$, a 1D-CNN applies a filter W by convolving across these embeddings as shown in Equation (2).

(2) $$Z_{j}=ReLU\left(W\cdot \left[v_{j};...v_{j+k-1}\right]+b\right)$$

where $\left[v_{j};...v_{j+k-1}\right]$ indicates concatenation of k adjacent embeddings, and b is a bias term. CNNs effectively capture local transitions between log events, by sliding across the sequence. After pooling these feature maps, a fully connected layer typically classifies whether an event window is anomalous. While CNNs can be computationally efficient, they are less effective in modeling temporal dependencies unless carefully engineered filters [22] or additional steps such as residual connections or multi-scale kernels [25] are employed.

#### 3.2.2. RNN-Based Detection Approaches

RNN and its variants, such as LSTM and GRU, are among the earliest deep architectures for capturing temporal dependencies. An LSTM, for instance, maintains hidden and cell states $h_{t}$ and $c_{t}$ and updates them at each time step t as shown in Equation (3):

(3) $$h_{t},c_{t}=LSTM\left(x_{t},\;h_{t-1},\;c_{t-1}\right)$$

where $x_{t}$ represents the embedding of the log event at time t. RNN-based detector flags deviations if observed events diverge significantly from expected patterns by predicting the anomaly likelihood of the next log key [28]. This approach captures longer-range dependencies compared to CNN-based approaches. However, as detailed in [7], anomaly manifestation in most publicly available log datasets is straightforward and can be easily signaled by specific keywords or short patterns. Therefore, a heavy sequence model such as LSTM or GRU might offer limited incremental gains, they might be less useful in datasets where anomalies appear as single-step events or have immediate, unambiguous signatures such as “Node down”, “Job failed with error code X”, etc. Furthermore, these models require a complex backpropagation through time, which can be unnecessarily resource-intensive when simpler detection models suffice.

#### 3.2.3. Attention-Based Detection Approaches

Transformers and self-attention models address the long-range dependency challenge by computing pairwise attention scores across the entire sequence [13,24,28]. For a given input, ${X\in R}^{nxd}$, attention is computed using key Query (Q), Key (K), and Value (V) matrices as given in Equation (4).

(4) $$Attention\left(Q,K.V\right)=SoftMax\;\left(\frac{QK^{T}}{\sqrt{d_{k}}}\right)V,$$

where $d_{k}$ is the dimension of the query/key vectors. Attention-based architecture can pinpoint crucial segments for anomaly detection by aggregating contextual relationships. However, (as discussed in Section 2), such complex models are overkill and resource-intensive for environments where anomaly detection is relatively simpler.

## 4. Statistical Analysis of System Log Datasets

**Figure 1:**
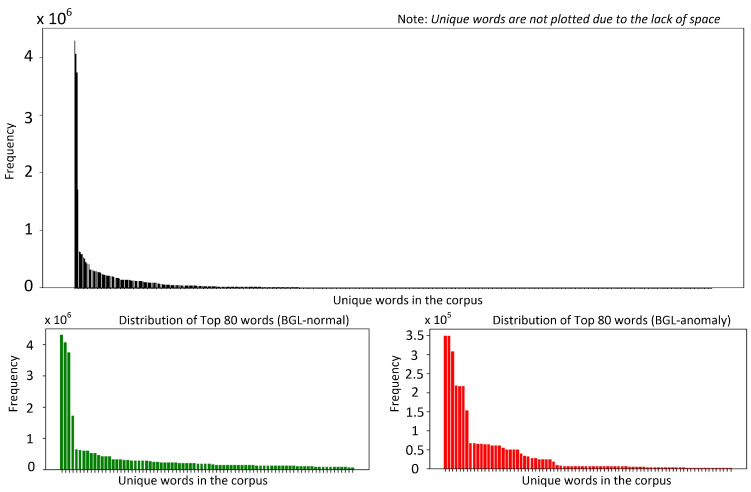
Word distribution (BlueGene/L (BGL) dataset).

**Figure 2:**
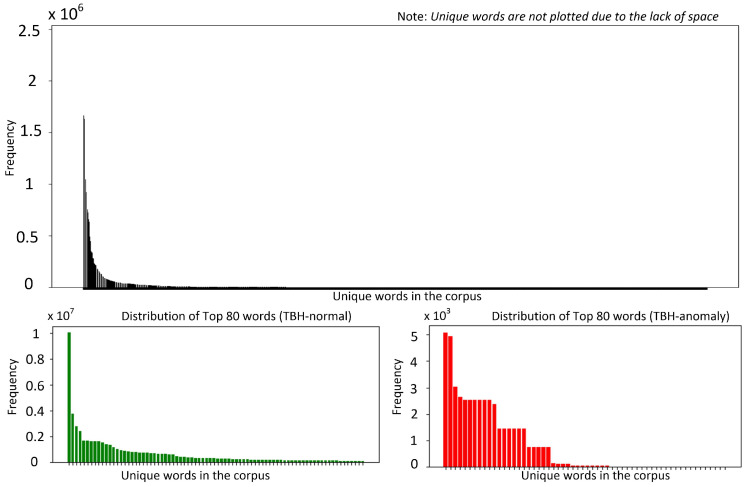
Word distribution (Thunderbird dataset).

Developing robust log anomaly detection systems requires a detailed understanding of the intrinsic properties of the datasets used for model training. This section explores the key characteristics of two widely utilized publicly available datasets, BGL [15] and Thunderbird [15], which serve as standard benchmarks in log anomaly detection research [7,14]. By examining their structure, distribution, and anomaly patterns, we establish a foundation for evaluating model performance and generalization capabilities across different log environments. Figure 1 and Figure 2 present token frequency histograms for the BGL and Thunderbird datasets, separately illustrating normal and anomalous logs. These visualizations highlight several critical characteristics that influence both model design and evaluation methodology. Both datasets exhibit highly skewed distributions, with a small subset of frequently occurring tokens accounting for a disproportionately large share of total occurrences. Such extreme concentration of token frequencies (approximated by heavy-tailed log-normal distributions) poses challenges for designing robust detection models, as many token representations are derived from limited examples. In logs from such environments, tokens often correspond to error codes, resource identifiers, or system calls, many of which appear sporadically, resulting in limited contextual information.

Moreover, the overlap in top-ranked tokens between the two datasets is minimal, especially within the anomalous logs. $\;T_{BGL}^{k}$ denotes the top k tokens in BGL dataset and $T_{THBRD}^{k}$ is denoted the top k tokens in the Thunderbird dataset, then $\left|T_{BGL}^{k}\cap T_{THBRD}^{k}\right|\ll k.$ k=2 for the normal dataset, while k=1 for the anomalous dataset. This discrepancy impedes straightforward cross-dataset generalization, since anomaly-indicating tokens in one environment may be entirely absent or extremely rare in the other. Finally, this skewed token coverage underscores the importance of domain adaptation. General-purpose pretrained language models can overlook rare tokens, especially if these tokens did not appear (or appeared infrequently) in mainstream corpora during pretraining. Domain-specific PLMs tackle the exhibited discrepancy by capturing specialized terminologies more effectively. However, they do not fully resolve the cross-domain adaptation problem as system reconfigurations and software patches quickly render older anomalies less relevant in a new environment.

## 5. Proposed Methods

Log anomaly detection in cloud computing environments requires an approach that can adapt to the semantic complexity of system logs, the dynamic nature of computational workloads, and the changing infrastructures at scale. The proposed method addresses these requirements through three main components: effective log preprocessing, domain-specific PLM-based embedding, and robust classification with a novel loss function. The primary design is motivated by the need to capture cloud-specific terminologies, retain essential temporal context, and attenuate the influence of outdated events.

### 5.1. Log Preprocessing

#### 5.1.1. Noise Reduction and Tokenization

System logs often contain extraneous elements such as numerical IDs, timestamps, or special symbols that contribute limited semantic value. To address this, the method initiates with noise reduction, removing purely numeric tokens (e.g., “1234”), non-informative punctuation (e.g., “:”, “=”), and operators (e.g., “<”, “>”) unless domain knowledge suggests they are meaningful indicators of anomalies. After removing non-semantic noise from the logs, WordPiece tokenization is applied to manage out-of-vocabulary (OOV) words. This process is particularly important in cloud computing environments, where domain-specific terminology and node-specific identifiers often appear in long or compound forms. Breaking these terms into subword units allows WordPiece to enhance vocabulary coverage and improve the representation of log data. After the initial cleaning and tokenization, each log entry is appended with special tokens ([CLS] at the start and [SEP] at the end) to present message boundaries for the downstream embedding model. When necessary, the sequence length is capped at 512 tokens to conform to the architectural constraints of the PLMs.

#### 5.1.2. Grouping and Temporal Ordering

To enable temporal context-aware training while maintaining the logical structure of log sequences, log entries are first grouped by their session ID and ordered chronologically based on their timestamps. This sequential arrangement is crucial for the subsequent loss function, where each event is assigned a time index t to control the decaying factor. In cloud computing environments, where logs originate from multiple nodes or compute jobs, this approach ensures a consistent temporal order, either globally or within job-specific execution traces, preserving coherence across distributed logging sources.

#### 5.1.3. Contextual Embedding Extraction

Motivated by [8], we adopted a domain-specific model named SecureBERT [29] to overcome the limitations of general-purpose PLMS (as discussed in Section 3). SecureBERT has been fine-tuned on a corpus of cybersecurity data. Such adaptation empowers the embeddings to better align with cloud computing-centric vocabularies, facilitating more accurate semantic representations. The model features 12 hidden layers, each with an output dimension of 768, 12 attention layers, and a feed-forward network size of 2048, with an input size of 512. Upon receiving a tokenized log message ${\left\{x_{i}^{T}\right\}}_{i=1}^{n};$ SecureBERT processes each token through multiple self-attention layers to generate contextual embeddings ${\left\{x_{i}^{e}\right\}}_{i=1}^{n}\in R^{d}.$ These embeddings capture both intra-log relationships, such as the relation of error codes to subsequent textual descriptions, and cloud-specific semantics. Additionally, following prior works in sequence classification [8], the final embedding ${x^{r}\in R}^{d}$ for each log message is obtained via an arithmetic mean of the last-layer token embeddings, as given in Equation (5).

(5) $$x^{r}=\frac{1}{n}\sum_{i=1}^{n}x_{i}^{e}$$

where d=768d (the hidden layer dimension). This strategy balances information retention with computational simplicity, yielding a fixed-size feature vector representing each log message.

### 5.2. Classification with Loss Incorporating Temporal Decay (LDF)

#### 5.2.1. Classification Head

After each log message has been converted to its 768-dimensional embedding, a fully connected neural network (FCNN) acts as the classification head, predicting the probability that the log message is anomalous. Concretely, the FCNN comprises two dense layers of sizes 64 and 32, each followed by a Rectified Linear Unit (ReLU) activation. The model size is chosen to minimize overfitting, as these logs can be highly repetitive and skewed. Furthermore, a neuron with a sigmoid activation (δ ∈[0,1]) is used in the output layer. The final output ${\hat{y}}_{t}$ serves as the anomaly score for the log at the time step t.

#### 5.2.2. Formal Definition of the Loss with Decaying Factor (LDF)

Let $y_{t}\in \{0,1\}$ be the true label for the log at time step t, and let ${\hat{y}}_{t}\in \{0,1\}$ be the classifier’s predicted probability of an anomaly; then, the binary cross-entropy loss at step t is calculated using Equation (6).

(6) $$L_{CE}\left({\hat{y}}_{t},y_{t}\right)=-\left[y_{t}\log\left({\hat{y}}_{t}\right)+\left(1-y_{t}\right)log\left(1-{\hat{y}}_{t}\right)\right]$$

The proposed Loss LDF modifies the total training objective to penalize older time steps with a factor $\alpha^{\left(t-t^{\prime }\right)}$, as given in Equation (7), where α∈[0,1] is a user-specified decay parameter and $t<t^{\prime }$ indexes historical events. Therefore, for each time step t the local LDF is

(7) $$L_{LDF}\left(t\right)=L_{CE}\left({\hat{y}}_{t},y_{t}\right)+\sum_{t^{\prime }\leq t}\alpha^{\left(t-t^{\prime }\right)}\;\cdot L_{CE}({\hat{y}}_{t^{\prime }},y_{t^{\prime }})$$

This ensures that recent events, ($t\approx t^{\prime }$), are more strongly weighted, whereas older ($t\ll t^{\prime }$) contributes exponentially less to the loss. By aggregating these weighted terms across the entire log sequence, as expressed in Equation (8), the final objective function for training is formulated as follows:

(8) $$L_{LDF}=\sum_{t=1}^{T}L_{LDF}(t)$$

where T denotes the total number of log messages in the training window.

#### 5.2.3. Choice of Decay Parameter α

The parameter α dictates how quickly historical anomalies lose impact. Empirically, α it may be tuned (e.g., in the range [0.90, 0.99]) based on validation performance. A smaller value rapidly reduces the influence of past events, making the model more reactive to recent anomalies. Conversely, a larger value retains a longer “memory” of the system state. In cloud computing environments, where failures often develop progressively, such as repeated “out of memory” logs leading up to a system crash, a balanced decay rate ensures the model neither disregards past patterns too quickly nor overemphasizes outdated information.

#### 5.2.4. Practical Implementation and Training

As each log message is processed at the time step t, the partial LDF penalty from all earlier steps is included. Although the backpropagation theoretically accumulates these weighted past cross-entropy terms, an efficient running sum is maintained in practice to prevent computational complexity from escalating exponentially. This optimization allows approximate gradient contributions from older events without excessive overhead.

Once trained, the model infers anomalies on a per-event basis using the sigmoid output ${\hat{y}}_{t}$. A threshold τ is applied to determine whether a log message is anomalous if ${\hat{y}}_{t}>\tau$. Adjusting τ allows operators to balance false positives and false negatives, ensuring adaptability to environmental and data variations. This final step helps the detection model adapt to environmental and data drift. Through jointly addressing heterogeneous cloud computing workloads (through domain-specific embeddings) and data drift (through decaying temporal dependency), the proposed approach aims to lower false positives and reinforce zero-shot performance.

## 6. Results

This section evaluates the proposed log anomaly detection method across various configurations and compares its performance to existing state-of-the-art approaches. Experiments are conducted using two well-known publicly available datasets, BGL [15] and Thunderbird [15], each with distinct statistical characteristics and operational patterns, as shown in Table 2. We implement the proposed method in Python (version 3.12) using PyTorch package (version 2.6). Experiments were conducted on a high-performance workstation running 64-bit Ubuntu 22.04.3 LTS OS (darkFlash Infotech Co., Ltd., Taipei, Taiwan), powered by an Intel Core (TM) i5-13400F 2.5 GHz with 128 GB RAM, and an NVIDIA RTX 4800 GPU with 16 GB RAM. Embeddings are extracted using the last encoder layer of SecureBERT [29], then averaged as expressed in Equation (5). The FCNN was trained for 30 epochs using the Adam optimizer (learning rate: 1 × 10^−4^) and a batch size of 32. The decay parameter α in LDF is empirically set in the range [0.9, 0.99] to balance historical context with rapid adaptation. We adopt F1-score, precision, recall, and false-positive rate to quantify model performance. $Precision=\frac{TP}{TP+FP\;}$, $Recall=\frac{TP\;}{TP+FN\;}$, $F1-score=2\left(\frac{Precision\;*\;Recall}{Precision+Recall}\right)$, where TP = true positive, FP = false Positive, and FN = false negative. In zero-shot experiments, we trained the model on BGL and tested it on Thunderbird, emphasizing the model’s ability to handle out-of-domain logs.

### 6.1. Effect of Domain-Specific Embedding

**Figure 3:**
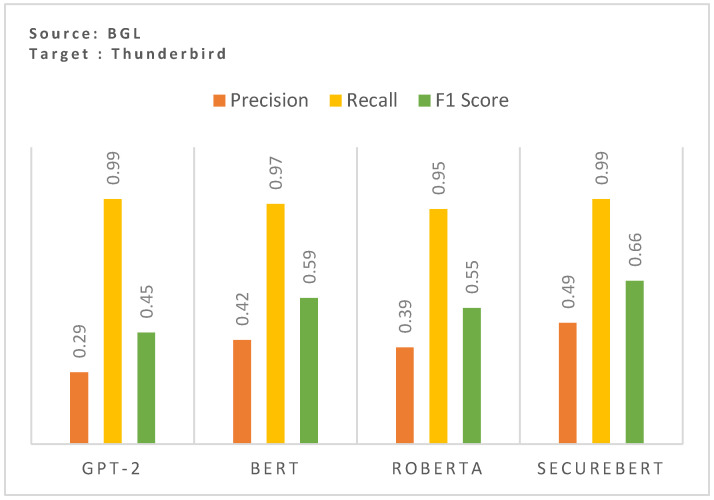
Effect of domain-specific embedding on the cross-domain zero-shot performance.

The results in Figure 3 highlight the impact of utilizing domain-specific embeddings on cross-domain zero-shot anomaly detection performance. The comparison between GPT-2, BERT, RoBERTa, and SecureBERT demonstrates a clear advantage in using domain-adapted embeddings when generalizing across different log datasets. The SecureBERT model achieves the highest F1-score (0.66), surpassing general-purpose models GPT-2 (0.32), BERT (0.29), and RoBERTa (0.35). This improvement is primarily due to the model’s enhanced ability to capture domain-specific terminology and structured log event patterns, enhancing the cross-domain performance.

RoBERTa performs slightly better in terms of precision (0.39). However, it still falls short of SecureBERT’s performance. These results emphasize that general-purpose PLMs struggle with log data due to its structured, domain-specific nature emphasizing the necessity of using a log-aware embedding space to enhance model robustness in cross-domain generalization.

### 6.2. Effect of the Time Decay Parameter α

**Figure 4:**
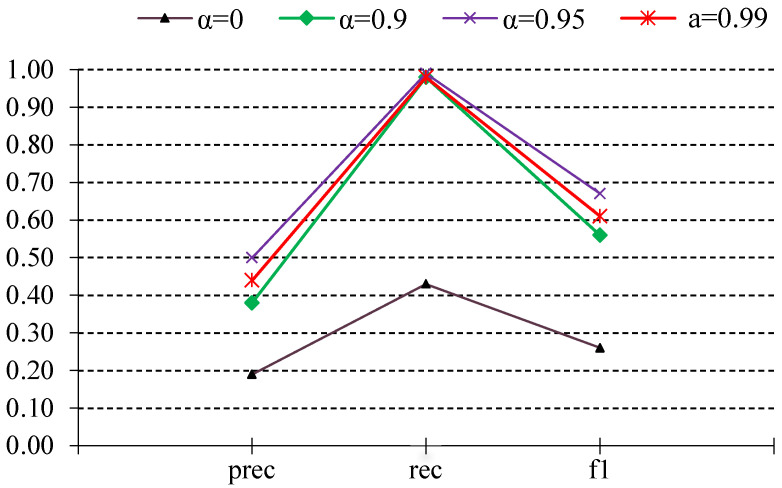
Effect of α parameter on the cross-domain zero-shot performance.

A distinguishing feature of our approach is the Loss with Decaying Factor (LDF), which modulates the contribution of older log events with the parameter α∈(0,1) governing the decay. We systematically vary α between 0.90 and 0.99 and record the resulting change in zero-shot detection performance as seen in Figure 4. Our empirical observations indicated a generally linear relationship between increasing α and improved detection performance, particularly in recall. Therefore, we selected the 0.90–0.99 range for detailed reporting, as it consistently delivered the best balance between historical awareness and real-time responsiveness. As α increases from 0.90 to 0.99, the model retains a longer memory of past anomalies, generally yielding a modest boost in performance as compared to α = 0. Too low a decay (e.g., 0.90) can lead the model to forget prior behavior too quickly, missing recurrent anomalies that emerge incrementally. Higher decay (0.95) helps capture slower-evolving anomalies. However, at the extreme end (0.99), older anomalies sometimes remain disproportionately influential, introducing noise into the decision boundary.

Moreover, this result suggests that while a slow decay rate retains more historical information, it may also reintroduce some outdated event influence, increasing false positives. This highlights the fundamental tradeoff between preserving historical context and avoiding over-reliance on past patterns in highly dynamic environments. Tuning α depends on the specific environment’s drift characteristics. However, in this case (α = 0.95) provides the best balance, ensuring that the most recent log sequences contribute strongly while irrelevant past anomalies lose significance. In an environment experiencing frequent reconfigurations or software updates, a lower α can prevent overfitting to old contexts. Conversely, environments exhibiting persistent, recurring faults may favor a higher value to preserve relevant historical patterns longer.

### 6.3. In-Domain Detection

The in-domain detection results in Table 3 highlight the effectiveness of the proposed method, which achieves competitive anomaly detection performance against state-of-the-art deep learning-based models, despite utilizing comparatively simpler architecture (simple MLP with LDF). On the BGL dataset, the proposed method attains an F1-score of 0.983, closely matching SaRLog [8] (0.988) and outperforming methods such as DeepLog [10] (0.930) and LogRobust [13] (0.753). Notably, while DeepLog achieves a perfect recall (1.000), its precision is lower (0.880), indicating a higher false positive rate. On the Thunderbird dataset, the proposed model continues to exhibit comparable performance achieving an F1-score of 0.941, outperforming SaRLog (0.999) and NeuralLog [23] (0.964). Compared to DeepLog (0.940), which also shows strong recall-based performance, the proposed method exhibits a better trade-off between anomaly sensitivity and specificity, reducing unnecessary false alarms.

Furthermore, it is important to highlight that the primary design objective of our model is to achieve robust generalization across both in-domain and cross-domain (zero-shot) scenarios. In contrast to the baseline methods that are primarily optimized for in-domain detection, our approach intentionally avoids overfitting to dataset-specific patterns. As a result, the relatively modest improvements in certain metrics reflect a deliberate trade-off—our method maintains strong in-domain performance while remaining adaptable to unseen log distributions. This balance between accuracy and adaptability is especially valuable in dynamic and evolving environments, such as cloud-based infrastructures. Additionally, these results support our core hypothesis that complex deep sequence models like LSTMs and Transformers are not strictly necessary for effective anomaly detection in structured log data. Our approach achieves competitive performance using a lightweight MLP combined with the proposed LDF mechanism. The LDF effectively captures evolving temporal dependencies without incurring the computational overhead typically associated with deep sequential architectures. This makes our method particularly well-suited for large-scale, real-time log analysis in environments where efficiency and scalability are critical.

### 6.4. Zero-Shot Performance

**Figure 5:**
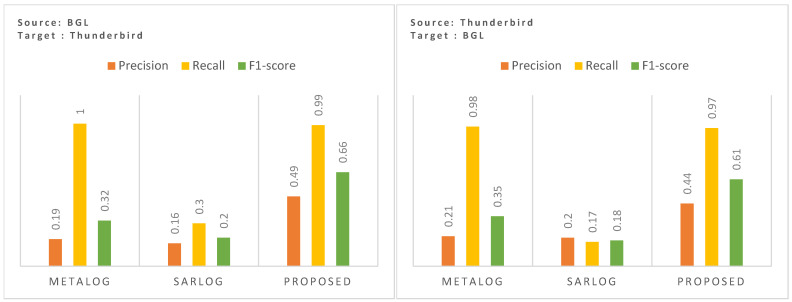
Cross-domain zero-shot performance, trained on BGL and tested on Thunderbird (**left**), trained on Thunderbird, and tested on BGL (**right**).

Zero-shot performance evaluation assesses a model’s ability to generalize across heterogeneous environments without retraining. We selected MetaLog [9] and SaRLog [8] as comparative baselines in Figure 5, as both models were explicitly designed to handle zero-shot and few-shot scenarios through meta-learning and contrastive learning techniques, respectively. This allows for a more meaningful and fair assessment of generalization performance. After replicating the model as described in both papers, the zero-shot performance was evaluated under two settings: first, when the models are trained on BGL and tested on Thunderbird, and second, when the models are trained on Thunderbird and tested on BGL.

As shown in Figure 5 (left), in our initial experiment (when the model was trained on BGL and tested on Thunderbird), MetaLog [9], while achieving a recall of 1.0, suffers from extremely poor precision (0.19), leading to a low F1-score of 0.32. This indicates that while MetaLog detects nearly all anomalies, it also generates a high number of false positives. SaRLog [8], on the other hand, shows slightly better precision (0.16) at the cost of a significantly lower recall (0.3), leading to a modest F1-score of 0.2. This suggests that SaRLog is more conservative in flagging anomalies, however, its lower recall makes it unsuitable for detecting rare but critical failures in a new dataset. In contrast, the proposed method achieves an F1-score of 0.66, significantly outperforming both baselines, reflecting the model’s robustness in maintaining high anomaly coverage while minimizing false positives.

To further evaluate bidirectional zero-shot capability, we conducted a reverse transfer experiment where the model is trained on Thunderbird and tested on BGL. The results, visualized in Figure 5 (right), indicate a similar trend. While MetaLog [9] and SaRLog [8] suffer from low generalization, the proposed method again achieves superior performance with a precision of 0.44, recall of 0.97, and F1-score of 0.61. This improvement is particularly notable given the significant domain shift and vocabulary divergence between Thunderbird and BGL datasets. Notably, MetaLog [9], despite achieving a high recall (0.98), falls short in precision (0.21), yielding a low F1-score (0.35). Similarly, SaRLog [8], while achieving precision and recall of 0.2 and 0.17, respectively, records the lowest F1-score (0.18). The proposed method, on the other hand, demonstrates a consistent performance boost, underscoring its cross-domain adaptability in both transfer directions.

The demonstrated results clearly indicate the adaptability of the proposed approach to the distributional shifts in log structures, terminology, and event frequency, making it a reliable solution for real-world zero-shot log anomaly detection in dynamic cloud environments. Furthermore, the substantial improvement in precision over MetaLog [9] and SaRLog [8] suggests that the proposed method does not overfit to dataset-specific patterns, instead leveraging a more adaptable temporal and semantic representation of log messages. This validates the hypothesis that integrating exponential time decay and domain-specific embeddings enhances anomaly detection, particularly in unseen datasets, making the approach a promising direction for robust cross-dataset log anomaly detection in cloud-centric environments.

## 7. Discussion

Our study underscores the importance of coupling domain-specific PLMs with temporal decay in log anomaly detection. LDF serves as a flexible and lightweight mechanism for integrating time-decay effects into the detection process. It offers a direct way to balance historical context with newly emerging evidence and is especially useful in dynamic environments such as supercomputing or cloud computing, where old information rapidly becomes outdated. In scenarios under which it is required to capture subtle, multi-step event patterns or cyclical phenomena, deep sequential models such as LSTMs may provide a richer, end-to-end temporal modeling approach. However, in more resource-constrained or rapidly shifting environments, utilizing LDF with simple and lightweight models may be preferable for its computational efficiency. Additionally, a hybrid approach combining sequential models with an LDF objective allows the model to learn temporal embeddings while still applying a decaying factor to fine-tune how it handles older events. The optimal choice ultimately depends on computational constraints, the complexity of temporal relationships within the log data, and the degree to which older events retain relevance in the target domain.

Moreover, we observe that statistically skewed vocabularies and class imbalances pose substantial barriers to robust cross-dataset generalization. While LDF partially mitigates this by focusing attention on newly emergent patterns in the test data, our current approach does not employ an explicit class weighting scheme. This design choice can lead to elevated false positives in datasets such as Thunderbird, where anomalies are vastly outnumbered by normal events. Furthermore, although exponential decay is computationally efficient and straightforward to implement, more sophisticated decay mechanisms with trainable α parameters might better capture evolving supercomputing log dynamics. Finally, exploring graph-based or hierarchical modeling could enhance detection accuracy in environments where logs arrive from multiple nodes or distinct job runs. These considerations highlight key opportunities to refine the proposed approach and reduce false positives in complex zero-shot scenarios.

## 8. Conclusions

This study presents a log anomaly detection method that combines a domain-specific PLM with a novel loss function, the Loss with Decaying Factor (LDF), to address the dual challenges of semantic complexity and evolving temporal patterns in cloud computing environments. Extensive experiments on the BGL and Thunderbird datasets demonstrate significant improvements in zero-shot generalization, thereby underscoring the robustness of the proposed approach in practical scenarios subject to continual drift and dataset variability. Our results suggest that future research might explore more adaptive decay models, incorporate more robust and lightweight sequence modeling architectures, and further tailor domain-specific PLMs to capture emerging domain terminologies. Overall, this work marks a significant step toward holistic, time-aware log anomaly detection that generalizes effectively across heterogeneous environments.

## Figures and Tables

**Table 1:** Summary of key related studies, their main objectives, and the core algorithms.

Paper	Objective	Algorithm/Method
DeepLog [10]	Real-time detection by modeling temporal dependencies at log-entry level	LSTM-based sequence modeling
LogAnomaly [11]	Improves detection with attention mechanism + template-based vectorization	RNN + attention for enhanced sequence modeling
LogRobust [13]	Captures bidirectional dependencies for robust detection	Bi-LSTM with attention
PLELog [27]	Detect log anomalies using a semi-supervised approach, estimating labels through probabilistic clustering.	Label Estimation using HDBSCAN clustering, Embedding with TF-IDF and FastText, and anomaly detection using Attention-based GRU.
LogAT [26]	Predict log anomalies in updated Hadoop systems by transferring knowledge from labeled legacy logs using unsupervised domain adaptation.	Adversarial transfer learning with CNNs for feature extraction and Bi-LSTMs for sequence modeling
NeuralLog [23]	Strengthens reliability and adaptability of detection	BERT-based representation + transformer-based classification
LAnoBERT [24]	Detect system log anomalies without relying on log parsers, leveraging a BERT-based masked language model.	BERT with Masked Language Modeling (MLM), Parser-free preprocessing with regular expressions, inference using a log dictionary database
SaRLog [8]	Few-shot learning with Siamese contrastive approach	BERT-augmented Siamese network + contrastive loss
MetaLog [9]	Zero-/few-shot cross-domain detection via meta-learning	Meta-learning + Globally Consistent Semantic Embedding (GCSE)

**Table 2:** Statistical information of the employed datasets.

Dataset	Log-Events	Training Data		Testing Data	
		Total	Alert	Total	Alert
BGL	1847	55,401	25,066	13,851	6309
Thunderbird	2880	400,000	193,840	100,000	3460

**Table 3:** In-domain detection performance of the proposed model.

Baseline Methods	BGL			Thunderbird		
	Precision	Recall	F1-Score	Precision	Recall	F1-Score
DeepLog [10]	0.880	1.000	0.930	0.940	0.941	0.940
LogRobust [13]	0.625	0.967	0.753	0.616	0.781	0.681
NeuralLog [23]	0.985	0.985	0.985	0.933	1.000	0.964
PLELog [27]	0.965	0.999	0.982	0.821	0.952	0.881
SaRLog [8]	0.994	0.982	0.988	1.000	0.999	0.999
Proposed Method	0.990	0.978	0.983	0.895	0.991	0.941

## Data Availability

The original data presented in the study are openly available in https://www.kaggle.com/datasets/omduggineni/loghub-bgl-log-data (Accessed on 06 April 2024).
